# Best Practices in Museum Programming for People With Dementia and Their Care Partners

**DOI:** 10.1093/geroni/igaa057.3061

**Published:** 2020-12-16

**Authors:** Carolyn Halpin-Healy, James Noble

**Affiliations:** 1 Arts & Minds, New York, New York, United States; 2 Columbia University Medical Center, New York, New York, United States

## Abstract

As more people are living longer with dementia, museums can play an important public health role to support individuals, families, and communities. Arts & Minds programs for people with dementia and their care partners are designed according to principles of person-centered care and adult learning theory. The approach builds on research and evaluation to provide contact with art, which has been shown to reduce apathy and soothe agitation in persons with dementia and also to reduce caregiver stress. According to our observation- and feedback-based assessments of more than 500 program participants over the past 10 years, the processes of collective looking and art making specifically address symptoms of dementia and support human dignity. This presentation addresses the growing research base on the benefits of museum programming, and the ways in which research and evaluation support best practices in museum education to address the challenge of living well with dementia.

